# Interpretable Machine Learning-Based Prediction Model for Concrete Cover Separation of FRP-Strengthened RC Beams

**DOI:** 10.3390/ma17091957

**Published:** 2024-04-23

**Authors:** Sheng Zheng, Tianyu Hu, Yong Yu

**Affiliations:** 1 School of Digital Construction, Shanghai Urban Construction Vocational College, Shanghai 201415, China; senblue@126.com; 2 School of Civil Engineering, Chongqing Jiaotong University, Chongqing 400074, China; 3 School of Civil Engineering and Engineering Management, Guangzhou Maritime University, Guangzhou 524088, China; yuyong1990@foxmail.com

**Keywords:** concrete cover separation, fiber-reinforced polymer, XGBoost, SHapley additive exPlanations

## Abstract

This study focuses on the prediction of concrete cover separation (CCS) in reinforced concrete beams strengthened by fiber-reinforced polymer (FRP) in flexure. First, machine learning models were constructed based on linear regression, support vector regression, BP neural networks, decision trees, random forests, and XGBoost algorithms. Secondly, the most suitable model for predicting CCS was identified based on the evaluation metrics and compared with the codes and the researcher’s model. Finally, a parametric study based on SHapley Additive exPlanations (SHAP) was carried out, and the following conclusions were obtained: XGBoost is best-suited for the prediction of CCS and codes, and researchers’ model accuracy needs to be improved and suffers from over or conservative estimation. The contributions of the concrete to the shear force and the yield strength of the reinforcement are the most important parameters for the CCS, where the shear force at the onset of CCS is approximately proportional to the contribution of the concrete to the shear force and approximately inversely proportional to the yield strength of the reinforcement.

## 1. Introduction

**Figure 1:**
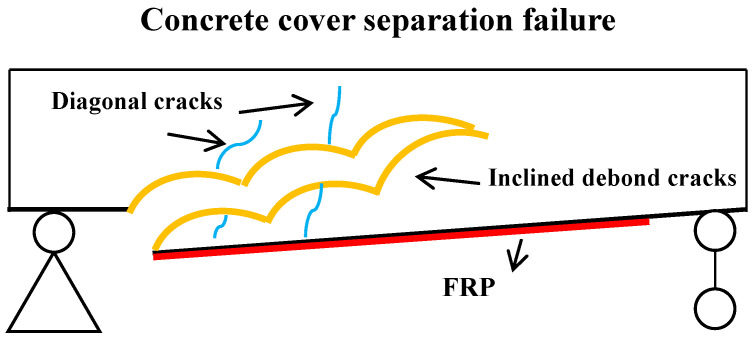
CCS failure.

In recent years, there has been a worldwide increase in the demand for upgrading and maintaining aging concrete structures [1]. Strengthening and repairing these structures have proven to be more cost-effective, time-efficient, and resource-friendly compared to replacing them entirely. Various strengthening techniques have been developed to effectively address the need for the strengthening and rehabilitation of structures [2,3]. One particularly popular solution for externally bonded reinforcement is fiber-reinforced polymer (FRP). FRP offers exceptional material properties including high tensile strength, stiffness, corrosion resistance, durability, low weight, ease of construction, and cost-effectiveness. These remarkable properties resulted in the widespread acceptance of FRP as an effective reinforcement solution [4,5,6,7]. Despite the advantages of FRP as an externally bonded strengthening material, the bond strength between the FRP and concrete substrate presents a significant challenge. This weak link in the system often results in premature failure of the FRP before it reaches its expected service life. Experimental studies have demonstrated several failure modes of FRP-strengthened concrete beams under flexural conditions, including intermediate crack (IC) debonding, plate-end (PE) interfacial debonding, concrete cover separation (CCS), concrete crushing, and FRP rupture. These failure modes highlight the complexity of the interaction between FRP and concrete, emphasizing the need for further research and development to address the bond strength issue and optimize the performance of FRP strengthening systems [8]. Two significant failure modes observed in FRP-strengthened concrete beams are IC debonding and PE debonding, the latter of which consists of plate-end interfacial debonding and CCS (Figure 1). Both IC debonding and PE debonding are commonly observed failures in FRP-strengthened concrete beams [9]. IC debonding in FRP-strengthened concrete beams is primarily caused by the development of flexural, shear, or flexural–shear cracks in the mid-span region, which exhibit geometric discontinuity. These cracks typically initiate at the point of higher bending moment (mid-span) and propagate toward the ends of the FRP plate, following the direction of decreasing bending moment. This behavior is commonly observed during IC debonding failure [10,11]. PE debonding is more likely to occur in FRP-strengthened concrete beams with a small shear span and where the mechanical behavior of the beam is primarily governed by shear forces instead of bending moments. In such cases, the small bending moments within the beam lead to a concentration of shear forces near the plate ends. This concentration of shear forces increases the likelihood of interfacial debonding between the FRP plate and the concrete, as well as the possibility of CCS [12]. This type of failure can manifest itself in two forms: CCS occurring at the height of the tensile reinforcement or plate-end interfacial debonding between the FRP plate and the concrete. In CCS, the concrete cover separates from the main body of the beam, while in plate-end interfacial debonding, the bond between the FRP plate and the concrete weakens or breaks. Among the two forms mentioned, CCS is generally observed to be more common than plate-end interfacial debonding [13,14]. CCS failure in FRP-strengthened concrete beams can be influenced by various factors such as concrete strength, concrete protective layer thickness, reinforcement ratio, FRP size, and modulus of elasticity [15,16,17]. When the FRP plate is positioned close to the support, CCS typically occurs after the development of shear cracks [18]. The loading process amplifies these shear cracks, thereby augmenting the interfacial shear stress. In addition, shear cracks often coincide with a relative vertical displacement between the two fractured surfaces. This displacement results in an increase in the transverse positive tensile stress within the concrete layer situated between the FRP and the tensile reinforcement. The combination of increased positive tensile stresses and interfacial stresses ultimately induces horizontal cracking and CCS at the precise horizontal location of the tensile reinforcement and concrete cover. When the plates are positioned away from the support in beams, CCS failure occurs following the development of diagonal cracks. These cracks initiate at the end of the plate and propagate toward the interior of the beam until they reach the location of the tensile reinforcement. Once they reach the horizontal position of the reinforcement, the cracks propagate horizontally, eventually leading to CCS failure [18,19].

Researchers and codes such as AS, ACI, TR55, and fib have proposed predictive models to estimate the failure loads of CCS [20,21,22,23,24,25]. These models are based on limited experimental data, and their accuracy remains to be verified. Al-Ghrery et al. developed a gene expression programming model for CCS prediction based on the constructed database. Compared with other models, it has higher goodness-of-fit and lower mean absolute error, but the accuracy and interpretability of the model need to be improved [26]. Based on this, a model with high accuracy and interpretability is still to be established.

This study attempts to construct the CCS prediction model based on interpretable machine learning [27,28]. Models including linear regression, support vector machine, BP neural network, decision tree, random forest, and XGBoost are constructed and compared with the models of codes and researchers, and finally the optimal model obtained is analyzed for interpretability using SHapley additive exPlanations (SHAP).

## 2. Workflow

This paper consists of the following four main sections:

Dataset construction: A total of 127 data sets were collected. The following parameters were included: beam effective depth (*d_s_*), beam width (*b*), concrete strength (*f′_c_*), area of the longitudinal reinforcement (*A_st_*), yield strength of the reinforcement (*f_sy_*), cross-sectional area of the FRP (*A_f_*), Young’s modulus of FRP (*E_f_*), ratio of the design moment to the design shear at the end of the FRP (M*/V*), contribution of the hoop reinforcement to the shear force (*V_us_*), and contribution of the concrete to the shear force (*V_uc_*). The output parameter is the shear force at the end of the FRP (V*) when CCS failure occurs.

Machine learning model construction: Prediction of concrete cover separation using six machine learning methods: linear regression, support vector machine, BP neural network, decision tree, random forest, and XGBoost.

Model evaluation: Evaluation of machine learning and existing models based on goodness of fit, root mean square error, and coefficient of variation.

Model explainability: Parameter importance and sensitivity analysis using SHapley additive exPlanations.

## 3. Dataset Construction

### 3.1. Parameter Selection Criteria

The performance of machine learning models is strongly influenced by the quality of the dataset, and this study constructs the dataset based on the following criteria:

(1) The failure mode of all beams is CCS, and there are no other modes.
(2) The geometrical characteristics and parameters of the beams are described in detail.
(3) The FRP sheets were not pre-stressed.

### 3.2. Inputs and Outputs

After researching the literature and data selection criteria based on Section 3.1, as shown in Table 1, a total of 127 experimental data sets from 36 researchers were collected for this study [8,12,14,29,30,31,32,33,34,35,36,37,38,39,40,41,42,43,44,45,46,47,48,49,50,51,52,53,54,55,56,57,58,59,60,61]. Detailed information on the parameters can be found in [26]. There are ten input parameters, which are beam effective depth (*d_s_*), beam width (*b*), concrete strength (*f′_c_*), area of the longitudinal reinforcement (*A_st_*), yield strength of the reinforcement (*f_sy_*), cross-sectional area of the FRP (*A_f_*), Young’s modulus of FRP (*E_f_*), ratio of the design moment to the design shear at the end of the FRP (M*/V*), contribution of the hoop reinforcement to the shear force (*V_us_*), and contribution of the concrete to the shear force (*V_uc_*). The output parameter is the shear force at the end of the FRP (V*) when CCS failure occurs.

### 3.3. Description of the Dataset

**Figure 2:**
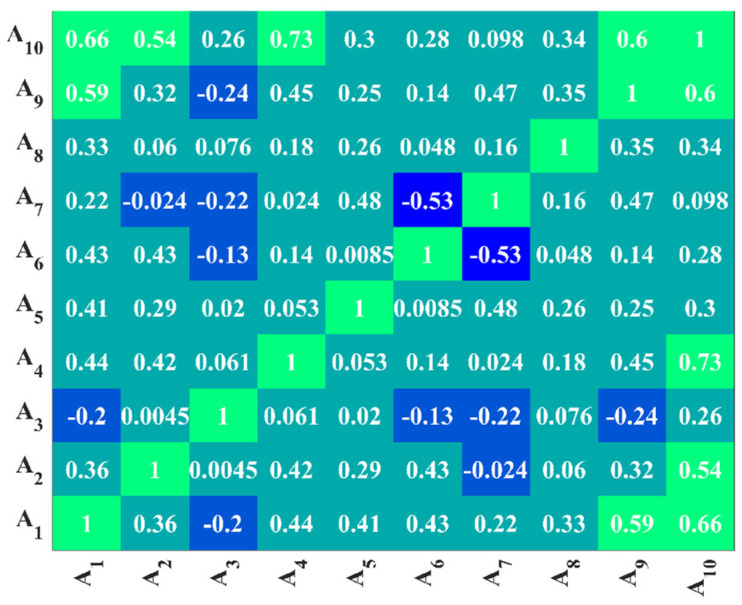
Correlation thermal Earth map of features.

In general, the performance of machine learning models is good when the distribution of the parameters in the dataset does not differ much from each other at intervals and the correlation between the parameters is weak, so the distribution and correlation of the parameters were investigated in this part. The distribution of the parameters is shown in Table 2, and the correlation heat map of the parameters is shown in Figure 2.

As can be seen from Table 2, the distributions of *A_st_*, *A_f_*, M*/V*, *V_us_*, and *V_uc_* are more concentrated, while the distributions of the other parameters are more discrete. From Figure 2, it can be seen that the correlation between most of the indicators is weak, except that there is a large correlation between A_1_ and A_9_ and A_10_. Therefore, the exclusion of A_1_ (*d_s_*) is taken into account in the modeling.

## 4. Machine Learning Models

### 4.1. Linear Regression

Linear regression (LR) modeling is a type of statistical model used to establish a linear relationship between variables [62]. It analyzes data by predicting the relationship between a dependent variable and one or more independent variables. Based on the method of least squares, the model parameters are estimated to minimize the difference between the predicted values and the actual observed values. The formula is as follows:

$$y=\beta_{0}+\beta_{1}x_{1}+\beta_{2}x_{2}+\ldots +\beta_{k}x_{k}+\varepsilon$$

where *y* is the predicted value, *β*_0_ is the intercept, *β*_1_ through *β*_k_ are the coefficients of the independent variables, and *ε* is the error term.

### 4.2. Support Vector Regression

Support vector regression (SVR) is a machine learning method for establishing the relationship between input variables and output variables [63]. The core idea of SVR is to find an optimal hyperplane in a high-dimensional space by using the principle of support vector machine (SVM), such that the interval between the training data points and this hyperplane is maximized. Unlike classification problems, the goal of SVR is to fit the training data as closely as possible given a certain tolerance and to minimize the error between the predicted results and the true values.

### 4.3. Backpropagation Neural Network

The backpropagation (BP) neural network model is an artificial neural network used to simulate the signaling process between neurons in the human brain. It consists of an input layer, a hidden layer, and an output layer, each of which contains multiple neurons, and the connections between neurons have weights that can be adjusted through learning [64]. The BP neural network trains the model by means of the backpropagation algorithm, which gradually reduces the error and improves the accuracy of the model by calculating the error between the output of the model and the actual output and propagating this error backward along the network in order to adjust the weights between the layers.

### 4.4. Decision Tree

Decision tree (DT) regression modeling is a machine learning method for establishing relationships between input variables and output variables [65]. Unlike decision trees in classification problems, decision tree regression models aim to predict continuous output variables rather than discrete labels. The model represents the relationship between data features and target variables by constructing a tree-like structure. Each node represents a feature; each branch represents a range of values for that feature, and the leaf nodes represent the predicted output values. The process of building a decision tree works by recursively partitioning the data set into subsets until a certain stopping condition is reached. One of the advantages of decision tree regression models is that they are easy to understand and interpret, as they generate models that can be visually represented as a series of simple rules.

### 4.5. Random Forest

Random forest (RF) regression modeling is an integrated learning approach to regression tasks based on an ensemble of decision trees. It improves the accuracy and stability of the model by constructing multiple decision trees and averaging or voting their outputs [66]. Random forests use data and feature randomization to create multiple decision trees, and by combining the outputs of multiple decision trees, random forests can better handle complex data relationships, reduce overfitting, and perform better with noisy data.

### 4.6. XGBoost

XGBoost (eXtreme gradient boosting) is a very popular machine learning algorithm for regression and classification tasks [67]. XGBoost is based on the gradient-boosting framework but uses a number of engineering optimizations to improve performance and accuracy. It is a decision tree-based model that predicts the values of continuous-type target variables by combining multiple decision trees. It does this by iteratively training weak classifiers (usually decision trees) and then adjusting subsequent classifiers based on the performance of previous classifiers to minimize the loss function. In this way, XGBoost is able to accumulate the predictive power of multiple models in an integrated model, thereby improving overall accuracy.

**Figure 3:**
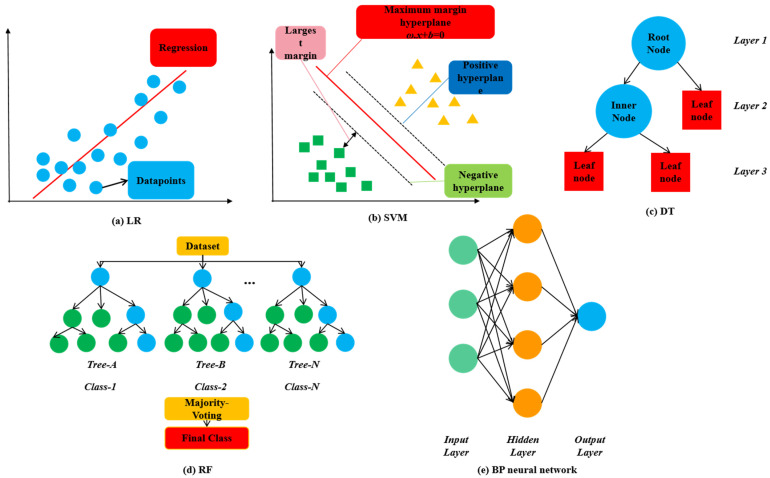
Machine learning models.

The above provides a brief overview of the machine learning methods used in this study (Figure 3); please refer to the literature [62,63,64,65,66,67] for further information.

### 4.7. Shapley Additive Explanation

Shapley additive explanation (SHAP) is one of the most popular model-agnostic methods available for enhancing the explainability of machine learning models [68]. Grounded in cooperative game theory, SHAP assigns feature importance using Shapley values. The Shapley value for a feature $\emptyset_{j}\left(val\right)$ is computed as the weighted sum of its marginal contributions across all possible feature subsets as shown in the equation below:

(1) $$\emptyset_{j}\left(val\right)=\sum_{S\subseteq \left\{1,\ldots ,p\right\}\setminus \left\{j\right\}}\frac{\left|S\right|!\left(p-\left|S\right|-1\right)!}{p!}\left(val\left(S\cup \left\{j\right\}\right)-val\left(S\right)\right),$$

where S is a feature subset, x  is the feature vector, and p  is the number of features. ${val}_{x}\left(S\right)$ represents the prediction for feature values in set S marginalized over features not included in set S:

(2) $${val}_{x}\left(S\right)=\int \hat{f}\left(x_{1},\ldots ,x_{p}\right)dp_{x\notin S}-E_{x}\left(\hat{f}\left(x\right)\right).$$

Averaging the absolute Shapley values across various instances, as illustrated in equation below, yields a more dependable measure of feature importance ($I_{j}$). This approach offers a thorough assessment of each feature’s impact on the model’s predictions, emphasizing features with higher absolute Shapley values as more impactful in the prediction process.

(3) $$I_{j}=\frac{1}{n}\sum_{i=1}^{n}\left|\phi_{j}^{\left(i\right)}\right|.$$

## 5. Results and Discussion

### 5.1. Machine Learning Model Construction

The six algorithms presented in Section 3 were used to construct the machine learning model. The data collected in Section 2 were used, of which 80% were used as the training set and 20% as the test set, and the hyperparameters of each model were determined by grid search and five-fold cross-validation.

### 5.2. Performance Criteria

The performance of the model was evaluated using the distribution of deviations between the predicted and true values, the goodness of fit (*R*^2^), and the root mean square error (*RMSE*), and the expressions for *R*^2^ and *RMSE* are as follows:

(4) $$R^{2}={\left[\frac{\sum_{i=1}^{k}\left(e_{i}-\bar{e_{i}}\right)\left(p_{i}-\bar{p_{i}}\right)}{\sqrt{\sum_{i=1}^{k}{\left(e_{i}-\bar{e_{i}}\right)}^{2}\sum_{i=1}^{k}{\left(p_{i}-\bar{p_{i}}\right)}^{2}}}\right]}^{2}$$

(5) $$RMSE=\sqrt{\frac{1}{N}\sum_{i=1}^{n}{\left(e_{i}-p_{i}\right)}^{2}}$$

### 5.3. Machine Learning Model Evaluation

**Figure 4:**
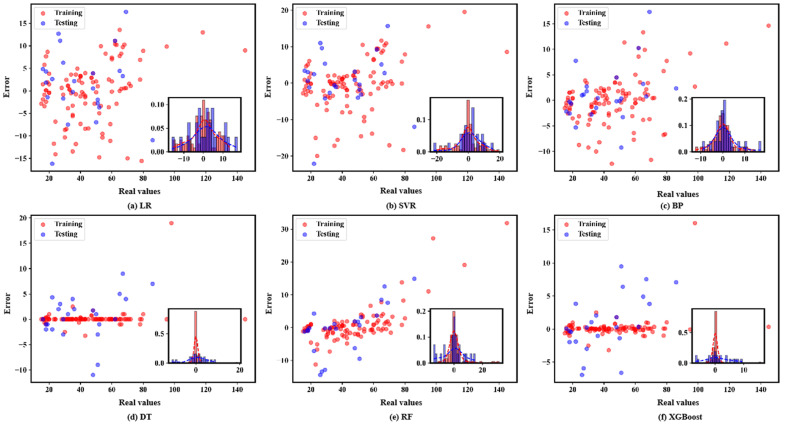
Deviations between predicted and real values.

The distribution of deviations between the predicted and true values of the six machine learning models are shown in Figure 4.

**Figure 5:**
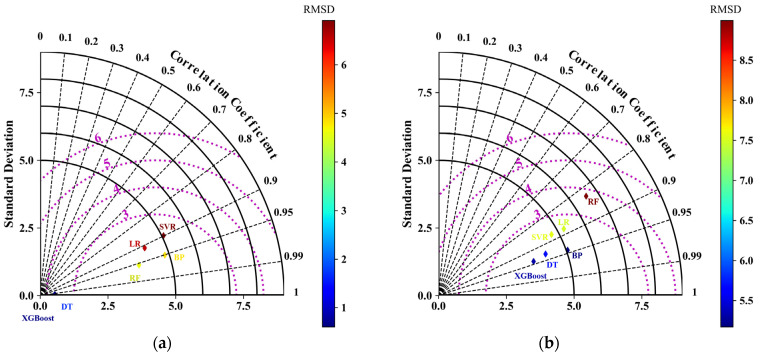
Taylor diagram of model performance for machine learning. (**a**) Training; (**b**) Test.

As can be seen from Figure 4, all machine learning models show better robustness, and their deviation distributions on both the training and test sets are approximately normally distributed. Among them, the bias distributions of DT and XGBoost are better than the other models. The above study shows the performance of each model in terms of deviation distribution. Considering that the deviation can only reflect the accuracy of the model to a certain extent, in order to further measure the performance of the model, Figure 5 further depicts the deviation, *R*^2^, and *RMSE* of each machine learning model on the training and test sets using Taylor diagrams.

As can be seen from Figure 5, XGBoost has the highest *R*^2^ and the lowest *RMSE* on both the training and test sets. LR, SVR, and RF have better performance on the training set, but perform poorly on the test set, and the generalization ability needs to be improved.

### 5.4. Existing Model Evaluation

**Figure 6:**
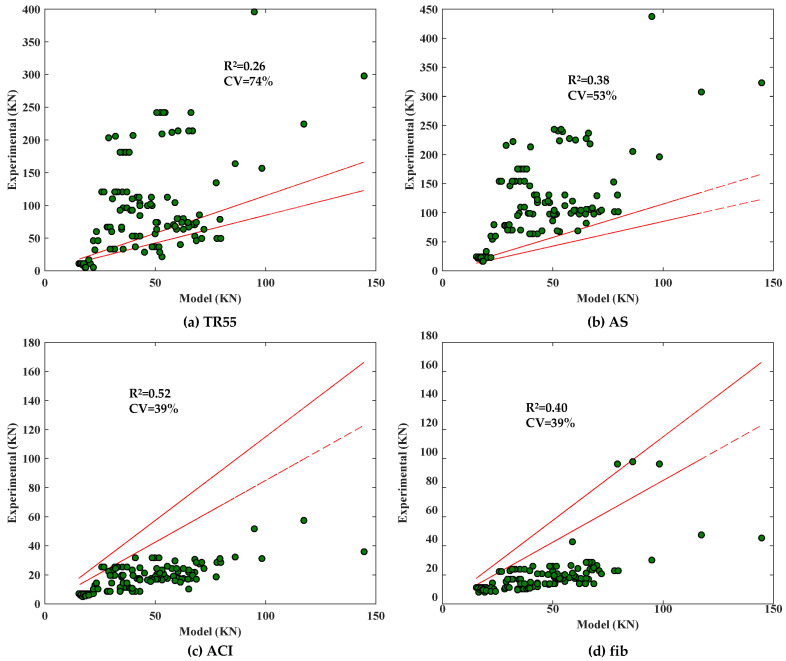
Performance of the model proposed by codes.

**Figure 7:**
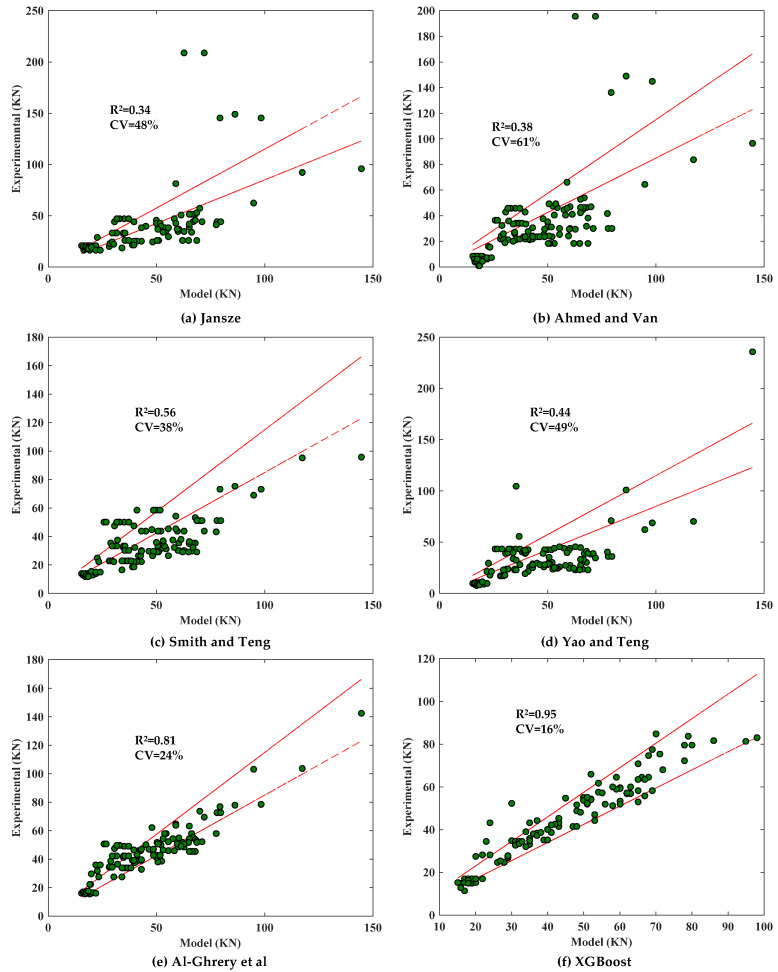
Performance of the model proposed by researchers [12,13,24,25,26].

The existing models for predicting CCS were analyzed using the database created in Section 2. The models were evaluated using the *R*^2^ and the coefficient of variation (CV), and the relationship between the calculated and experimental values of the code-suggested models is shown in Figure 6, and the relationship between the calculated and experimental values of the researcher-suggested models is shown in Figure 7.

As can be seen from Figure 6, the model proposed by ACI performs well, but both it and the model of fib are conservative; in addition, the models proposed by TR55 and AS overestimate the shear force at the end of the FRP sheet when CCS occurs. As can be seen from Figure 7, the XGBoost model developed in this paper has the highest *R*^2^ (0.95) and the lowest CV (16%), which is better than the models proposed by the researchers and codes in Figure 6 and Figure 7.

## 6. Parametric Study

**Figure 8:**
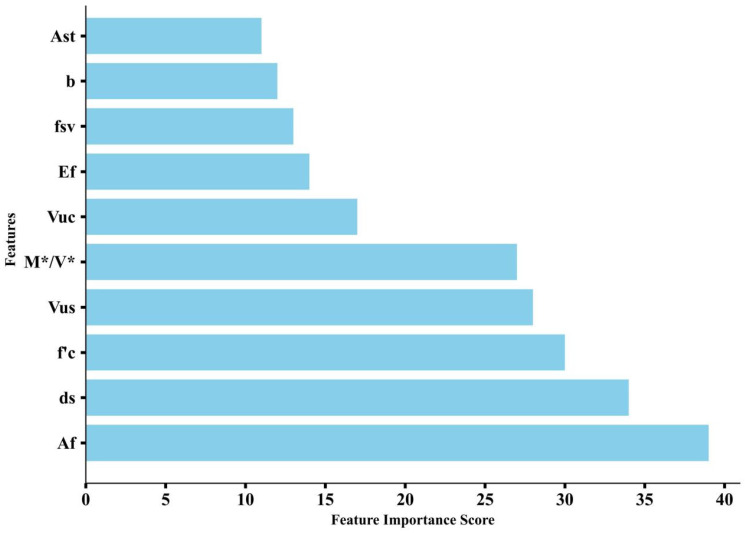
Importance of parameters based on XGBoost.

**Figure 9:**
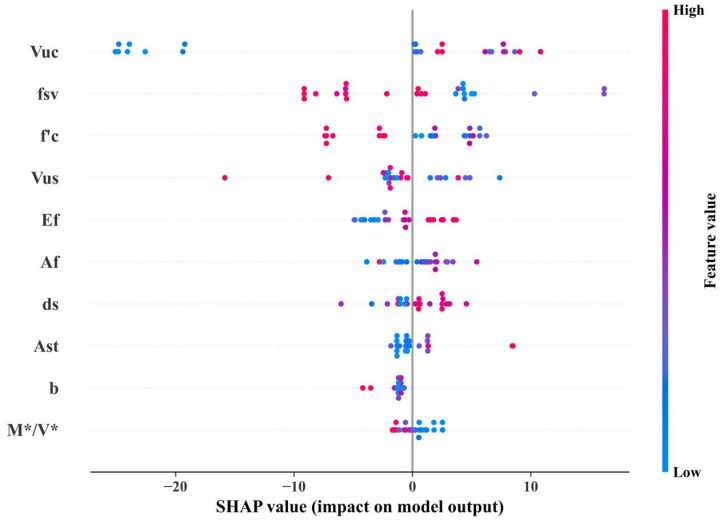
Importance of parameters based on SHAP.

As can be seen in Section 4.3, XGBoost performs best for all the machine learning models. It is therefore used for parametric studies. Two methods were used, one based on the importance ranking of the parameters themselves during the model training process, and one based on the model interpretable analysis of SHapley additive exPlanations (SHAP). The former is shown in Figure 8, and the latter is shown in Figure 9.

**Figure 10:**
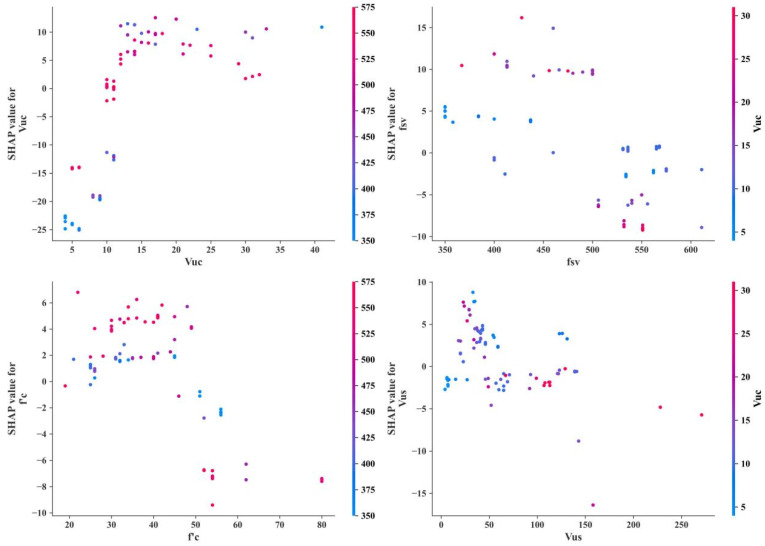
SHAP-based parameter sensitivity study.

As can be seen from Figure 8 and Figure 9, there is a large difference between the XGBoost-based parameter importance ranking and the SHAP-based results. However, they both consider that *f′_c_* and *V_us_* have a large effect on V*, and *A_st_* and *b* have a smaller effect on V*. The sensitivities between each parameter and V* are also shown in SHAP, which is shown in Figure 10.

Figure 10 shows the relationship between the four main parameters and V*. It can be seen that when *V_uc_* is small (<15), *V_uc_* and V* have an approximately linear relationship, but as *V_uc_* increases, it does not cause a significant change in V*. There is an inverse relationship between V* and *f_sv_*, but the trend is not significant. In addition, when *f′_c_* is small (<50 MPa), the relationship between V* and *f′_c_* is not obvious, and when *f′_c_* is large, as *f′_c_* increases, V* decreases. There is no obvious relationship between *V_us_* and V*.

## 7. Conclusions

In this study, a prediction model for the concrete cover separation in FRP flexure-strengthened RC beams based on machine learning was evaluated against the codes and the researcher’s model. Finally, a parametric study based on SHAP was carried out. The following conclusions were obtained:

(1) Of all the machine learning models, XGBoost is the best at predicting CCS, with a better distribution of deviations on both the training and test sets. In addition, the XGBoost model also has the maximum goodness-of-fit, the minimum standard deviation, and the minimum root mean square error on both the training and test sets.
(2) The models proposed by AS and TR55 overestimated the shear force during CCS, while the models of ACI, fib, and most researchers are conservative. In addition, the *R*^2^ and CV of these models are not satisfactory. Compared to the above models, XGBoost has a higher *R*^2^ (0.95) and a lower CV (16%).
(3) The parameters that have a greater influence on V* are the contribution of the concrete to the shear force, the yield strength of the reinforcement, the concrete strength, and the contribution of the hoop reinforcement where V* is approximately proportional to the contribution of the concrete to the shear force and approximately inversely proportional to the yield strength of the reinforcement and the concrete strength.
(4) In this study, the parameters affecting CCS failure were statistically analyzed based on SHAP. However, mechanism-based analyses are scarce and further research is needed in the future.

## Figures and Tables

**Table 1:** Data sources.

Reference	Number	Reference	Number
[8]		[44]	4
[12]		[45]	1
[14]	5	[46]	1
[29]	4	[47]	3
[30]	10	[48]	6
[31]	5	[49]	5
[32]	4	[50]	1
[33]	5	[51]	3
[34]	1	[52]	1
[35]	3	[53]	3
[36]	4	[54]	2
[37]	4	[55]	1
[38]	1	[56]	1
[39]	1	[57]	2
[40]	3	[58]	1
[41]	6	[59]	2
[42]	3	[60]	1
[43]	3	[61]	6

**Table 2:** Statistical values for input.

Parameter	*d_s_* (A_1_)	*B* (A_2_)	*f′_c_* (A_3_)	*A_st_* (A_4_)	*f_sy_* (A_5_)	*A_f_* (A_6_)	*E_f_* (A_7_)	M*/V* (A_8_)	*V_us_* (A_9_)	*V_uc_* (A_10_)
Min.	69	100	19	57	350	13	10	0	3	4
Max.	375	400	80	1272	611	912	271	550	491	182
Average	176	139	42	224	481	120	185	128	89	34
Standard deviation	47%	35%	53%	18%	79%	13%	68%	23%	18%	19%

## Data Availability

The raw data supporting the conclusions of this article will be made available by the authors on request.
